# Organic and Social Heredity

**Published:** 1915-03

**Authors:** 


### The Department of Eugenics, Mental and Social Hygiene

CONDUCTED BY
DR. MALONE DUGGAN
813-814 Gibbs Building, San Antonio, Texas

All communications for this department should be sent to DR. DUGGAN

### Organic and Social Heredity.

Much confusion exists among many today regarding the inheritance of acquired characters. We are told that acquired characters are not transmitted by heredity, that only fixed racial units are so inherited. This opens the question, then, as to how cultural progress is handed down from generation to generation. We see the material evidence of this in the advancement of science and art, in invention and commerce, and wonder how this can be, if the acquirements and accomplishments of the preceding generation are not inherited. The explanation lies in the fact of there being two kinds of inheritance. Organic heredity, which transmits only fixed racial characteristics, and social heredity, which transmits the cultural development, are acquired characters of the race. Man inherits the basic principles of the species through his organic heredity and on this substrata he builds his social life. In the first kind only the fixed heritable or germinal units are transmitted, in the other only the acquired characters.

In the long ago, in the evolution of man, there were certain instincts that led to a social life and self-sacrifice which in time resulted in a social heredity. Through natural selection his nervous system became more complex and composed of fewer fixed characters. For this reason he became more plastic and capable of greater moulding. With the animal, on the other hand, there has not been this distinction; its nervous system has developed only the fixed traits and therefore it has been left to survive by its natural instincts. This explains why the laws governing animal breeding cannot be applied to human betterment. It also makes clear the meaning of the eugenists when they state that the race has not improved innately for the past two thousand years. It is organic heredity that is understood here and not social. Indeed, the race is degenerating for the very reason that social evolution has advanced so much more rapidly than has organic evolution, and places a greater burden on the present generation than was possible in the earlier ages.

